# Higher serum 25-hydroxyvitamin D concentrations are associated with active pulmonary tuberculosis in hospitalised HIV infected patients in a low income tropical setting: a cross sectional study

**DOI:** 10.1186/s12890-018-0640-6

**Published:** 2018-05-08

**Authors:** Cuthbert Musarurwa, Lynn Sodai Zijenah, Doreen Zvipo Mhandire, Tsitsi Bandason, Kudakwashe Mhandire, Maria Mary Chipiti, Marshall Wesley Munjoma, Witmore Bayayi Mujaji

**Affiliations:** 1 Department of Chemical Pathology, University of Zimbabwe, College of Health Sciences, P.O. Box A178, Avondale, Harare Zimbabwe; 2 Department of Immunology, University of Zimbabwe, College of Health Sciences, P.O. Box A178, Avondale, Harare Zimbabwe; 3 Biomedical Research and Training Institute, Harare, Zimbabwe; 4 Department of Obstetrics and Gynaecology, University of Zimbabwe, College of Health Sciences, P.O. Box A178, Avondale, Harare Zimbabwe

**Keywords:** Vitamin D deficiency, Pulmonary tuberculosis, HIV, Hospital stay, cART

## Abstract

**Background:**

The inherent risk of developing tuberculosis (TB) in HIV- infected individuals is further enhanced by hypovitaminosis D. Interventions that offset HIV-associated immune deterioration potentially arrest disease progression and incidence of opportunistic infections including TB. Despite conflicting reports on association between vitamin D deficiency (VDD) and risk of TB, vitamin D (VD) supplementation remains a promising intervention.

**Methods:**

We conducted a comparative cross-sectional study on 145 HIV^+^/pulmonary TB^+^ (PTB) and 139 HIV^+^/PTB^−^ hospitalised patients to investigate association of vitamin D status and risk of PTB. Stratified random sampling was used to select archived serum specimens from participants enrolled in a randomised controlled trial (RCT) conducted to investigate the impact of using a point-of-care urine lipoarabinomannan strip test for TB diagnosis. PTB status was confirmed using sputum smear microscopy, culture or GeneXpert MTB/RIF. Serum 25-hydroxyvitamin D [25(OH) D] concentrations were assayed by competitive chemiluminescent immunoassay prior to commencement of anti-TB treatment. Effect of VD status on duration of hospital stay and patient outcomes on follow up at 8 weeks were also investigated. Median serum 25(OH) D concentrations were compared using Mann-Whitney test and covariates of serum VD status were assessed using logistic regression analysis.

**Results:**

Overall VDD prevalence in the cohort was 40.9% (95% CI: 35.1–46.8). Median serum 25(OH)D concentrations were significantly higher in HIV^+^/PTB^+^ group (25.3 ng/ml, IQR:18.0–33.7) compared to the HIV^+^/PTB^−^ group (20.4 ng/ml, IQR:14.6–26.9), *p* = 0.0003. Patients with serum 25(OH) D concentration ≥ 30 ng/ml were 1.9 times more likely to be PTB^+^ compared to those with serum 25(OH) D concentrations < 30 ng/ml (odds ratio (OR) 1.91; 95% CI 1.1–3.2). PTB-related death was associated with higher odds of having 25(OH) D levels≥30 ng/ml. Age, gender, CD4^+^ count, combination antiretroviral therapy (cART) status, efavirenz based cART regimen and length of hospital stay were not associated with vitamin D status.

**Conclusions:**

The finding of an association between higher serum 25(OH) D concentrations and active PTB and TB-related mortality among hospitalised HIV-infected patients in the present study is at variance with the commonly reported association of hypovitaminosis and susceptibility to TB. Our findings though, are in concordance with a small pool of reports from other settings.

## Background

Zimbabwe, ranked 13th of 22 high TB burden countries that account for 80% of global TB burden has an HIV co-infection rate of approximately 68% [1]. In a life-time, 10% of immunocompetent individuals asymptomatically infected with *Mycobacterial tuberculosis* (MTB), progress to active TB compared to 10% per year of HIV-infected individuals [2].

In vitro studies suggest vitamin D supplementation minimises progression to active TB given the role of vitamin D in immune regulation [3, 4]. These studies reported further an association between hypovitaminosis D with decreased macrophage activation and suboptimal production of cathelicidin both of which blunt the host’s ability to fight TB and other infections. One billion people worldwide are estimated to be vitamin D deficient [5]. HIV- infected individuals face a higher risk of hypovitaminosis D due to a unique risk profile associated with treatment or mere infection itself thus further enhancing susceptibility to TB [6]. HIV infection itself has been shown to increase metabolism of 25(OH) D into the active form, further driving vitamin D deficiency, due to gp120 induced CYP27B1 production [7].

Although some studies have associated VDD with poorer HIV infection outcomes and disease progression, in both cART-naive and treated patients [8, 9], the evidence for the association between VDD and increased TB risk is not sufficiently robust [10–12]. Clinical trials using 25(OH) D supplementation as adjunctive TB therapy reported no clinical benefits [13, 14] casting further doubt on the implied role of vitamin D on antimicrobial immunity. A study from The Gambia reported significantly higher median serum 25(OH) D concentrations in TB cases compared to tuberculin skin test positive controls and household contacts [10]. Another study conducted in Tanzania reported higher mean serum vitamin D levels in PTB patients compared to healthy controls but no significant difference between confirmed PTB^+^ patients and PTB suspects who tested negative for PTB [15]. Both high and low serum vitamin D concentrations were associated with susceptibility to PTB in Greenlanders [16]. Furthermore, a meta-analysis concluded that the association between VDD and increased risk of TB was lacking in HIV-infected or -uninfected African populations [12].These conflicting findings may be attributed to underlying genetic differences, diet, exposure to sun or skin colour.

There is paucity of research on the relationship between vitamin D status and HIV-related outcomes, particularly in resource-limited settings where both HIV and TB are co-endemic. Interventions that offset immune deterioration associated with HIV infection can potentially arrest disease progression and incidence of opportunistic infections including TB. We examined the prevalence, covariates of VDD and risk of PTB among HIV- infected hospitalized patients with and without PTB.

## Methods

### Study setting and design

The study was conducted at Mabvuku Polyclinic and Parirenyatwa Group of Hospitals, Harare, Zimbabwe. Harare is located at an altitude of 1480 m, latitude 17°55’S and longitude 31°7′E. The mean daily sunshine duration is about 8.2 h with the rainy season stretching from November to April. Study participants were enrolled from January 2013 to September 2014.

Two hundred and eighty four hospitalised, Black Zimbabwean patients comprising 145 HIV infected patients with active PTB (HIV^+^/PTB^+^) and 139 HIV infected patients without active PTB (HIV^+^/PTB^−^) were randomly selected from a cohort (*n* = 920) who participated in a randomised controlled trial (RCT) entitled ‘A RCT to evaluate the impact of using a point-of-care urine lipoarabinomannan (LAM) strip test for TB diagnosis amongst hospitalized HIV-infected patients in resource-poor settings (RCT LAM)’ whose details are described elsewhere [17, 18]. Briefly, to be eligible for inclusion into the RCT, potential participants were required to be HIV infected, presenting with any one of TB symptoms (fever, cough, drenching night sweats or self-reported weight loss) and sufficiently ill to warrant hospitalization. Patients demographic, clinical and laboratory data were abstracted from the RCT LAM database. Such data included length of hospital stay, and mortality outcomes 8-weeks post enrolment. Cause of death was recorded as tuberculosis, or not tuberculosis with the alternative cause of death specified. All data and specimens were anonymized before access was allowed to the current investigators.

### Study patients

Consenting study patients aged ≥18 years were all dark skinned, unveiled, with faces and arms regularly exposed to the sun. All were indigenous Zimbabweans, HIV- infected and suspected of having TB. Participants from the RCT-LAM study were stratified by PTB status before stratified randomisation was used to select participants from the RCT-LAM study. Participants who had been immobilised by illness for more than 2 weeks were excluded as were those with liver or renal conditions. Individuals who had already commenced anti-TB treatment and those on vitamin supplements were also excluded. The estimated sample size was 138 participants for each of the PTB status strata.

### Laboratory tests

Blood specimens were collected for enumeration of CD4+ T-lymphocytes, haemoglobin and plasma and serum processing within 6 h of venepuncture before participants commenced anti-TB treatment.

At enrolment, each patient submitted at least three sputum specimens utilised as follows (i) same day PTB testing (Xpert Mycobacterium tuberculosis/rifampicin (MTB/RIF) Version G4 and fluorescence smear microscopy) (ii) TB culture and (iii) archived for future studies. Sputum smear fluorescence microscopy, Xpert MTB/RIF assay and the Mycobacteria Growth Indicator Tube (MGIT BD Microbiology Systems, Cockeysville, MD, USA) culture to confirm PTB, were performed as previously described [17, 18].

### 25(OH) D assays

Serum specimens archived at -80 °C were retrieved and thawed only once before 25(OH) D assays were carried out. Serum 25(OH) D was measured as a marker of vitamin D status using a fully automated competitive chemiluminescent immunoassay analyser (Maglumi 2000 Snibe Co. Ltd. Shenzhen, 518,057 China) following manufacturer’s instructions. Between-run precision coefficients of variation for the assay ranged from 6.04 to 6.25% and within run precision coefficients of variation ranged from 3.01–3.45%.

Serum 25(OH) D concentrations reflect overall VD status because of a longer half-life of approximately 15 days compared to 15 h for the active form [19]. VDD was defined as serum 25(OH) D concentration < 20 ng/mL, vitamin D insufficiency as serum 25(OH) D concentration of 20–29 ng/mL, and sufficient vitamin D status as serum 25(OH) D ≥ 30 ng/ml [20]. Finally, 25(OH) D concentrations below 10 ng/ml were classified as severe VDD [21]. From these definitions, we further generated other dichotomies of vitamin D status as follows, optimal or suboptimal (≥ 30 ng/ml or < 30 ng/ml) and deficient or not deficient (< 20 ng/ml or ≥ 20 ng/ml).

### Ethics statement

The study protocol was approved by the Medical Research Council of Zimbabwe (MRCZ/A/1906). All the patients in the parent study (RCT-LAM) consented to future use of their left-over archived specimens and data in future TB- and/or HIV-related studies.

### Statistical analyses

Statistical analyses were conducted using STATA (version 13.0; Stata Corporation, College Station, Texas, USA). Counts and proportions (%) were used to summarise data. Medians and interquartile range (IQR) were used to summarise non-normally distributed variables and means ± standard deviations (SD) for normally distributed data. Differences in proportions were tested using the Chi-square test whilst differences between groups of continuous normally distributed data were tested using independent samples t-test and Mann-Whitney test for non-normal data. The prevalence of VDD was estimated in the whole sample and for the sub-groups (PTB^−^ and PTB^+^) with 95% confidence intervals (95% CI). Logistic regression was used to identify factors associated with VDD with OR and 95% CI were reported for all such cases. For all statistical comparisons α was set at 0.05.

## Results

Baseline clinico-demographic characteristics of the patients are presented in Table 1.

**Table 1:** Participants clinico-demographic data

Variable	All *n* = 284	HIV^+^/ PTB^+^ *n* = 145	HIV^+^/ PTB^−^ *n* = 139	*p* - value
Age median(IQR)	38 (32–46)	38 (32–44)	38 (32–47)	0.534
Female	142 (50)	72 (49.6)	70(50.4)	0.893
On cART	140(49.3)	62 (47.3)	78(56.1)	0.138
Duration of cART (months) Median(IQR)	21 (1–58)	10 (1–48)	32 (9–58)	0.076
Alive at Week-8 Follow-up	191(70.7)	90(62.1)	101(72.7)	0.057
Duration of Hospital Stay (Days) Median(IQR)	5(3–8)	4(3–7)	5(4–10)	< 0.001
CD4 T-lymphocytes count (cells/μL) median(IQR)	54(18–144)	41 (14–110)	86(25–220)	< 0.001
BMI Median(IQR)	19.2(17.0–21.5)	19.1(16.6–20.8)	19.2(17.3–21.8)	0.222
BMI ≤18.5	95(33.4)	48(33.1)	47(33.8)	0.901
On efavirenz based regimen	109(38.3)	48(33.1)	61 (43.9)	0.061
Haemoglobin g/dl mean(SD)	9.2(2.8)	8.6(2.4)	9.6(3.0)	0.030

Key: *n* = number of participants in category, *IQR* interquartile range, *SD* standard deviation, *BMI* Body Mass Index, *cART* combination Antiretroviral therapy, All values are stated as n(%) unless indicated otherwise. *p*-values of ≤ 0.05 were considered statistically significant

Of the 284 patients, 142(50%) were female with median age 38 (IQR32–46) years. There was however no significant difference in gender (*p* = 0.893), age (*p* = 0.534) and duration of cART (*p* = 0.076) between HIV^+^/PTB^+^ and HIV^+^/PTB^−^ groups. Of 140 patients on cART, 109(77.9%) were on an efavirenz (EFV)-based cART regimen.

There was however, no significant difference in proportions of patients on EFV-based regimen between the two groups (*p* = 0.061). At 8-week follow-up, there was no significant difference in mortality rates (*p* = 0.057) between the two groups although the HIV^+^/PTB^−^ patients stayed significantly longer in hospital compared to the HIV^+^/ PTB^+^ (*p* < 0.001). Median CD4+ T- lymphocyte counts were significantly lower in HIV^+^/ PTB^+^ patients (*p* < 0.001) as was mean haemoglobin concentration (*p* = 0.03).

The median serum 25(OH) D concentrations and vitamin D status for all participant groups are presented in Table 2.

**Table 2:** Median 25(OH) D concentrations and vitamin D status

Variable	All *n* = 284	HIV^+^/PTB^+^ *n* = 145	HIV^+^/PTB^−^ *n* = 139	*p*-value
Serum 25(OH) D ng/ml Median (IQR)	22.1 (16.4–31.5)	25.3 (18.0–33.7)	20.4 (14.6–26.9)	0.0003
Vitamin D Deficient	116(40.9)	49 (33.8)	67(48.2)	0.014
Vitamin D Insufficient	88(31)	46 (31.7)	42(30.2)	0.790
Optimal Vitamin D	80(28.2)	50(34.5)	30(21.6)	0.016
Severe vitamin D Deficiency	5 (1.8)	0(0)	5(3.6)	0.021

Key: Vitamin D deficient: 25(OH)D: < 20 ng/ml, Vitamin D insufficient: 25(OH) D: 20-29 ng/ml, Optimal Vitamin D 25(OH) D: ≥ 30 ng/ml, Severe Vitamin D Deficiency: 25(OH) D: < 10 ng/ml, *n* number of patients in each category. All values expressed as *n*(%) unless otherwise stated. *p*-values of ≤ 0.05 were considered statistically significant

Overall median serum 25 (OH) D concentration was 22.1 ng/ml (IQR16.4–31.5). The HIV^+^/PTB^+^ group had significantly higher median serum 25(OH) D concentration 25.3 ng/ml (IQR18.0–33.7) compared to the HIV^+^/ PTB^−^ group 20.4 (IQR14.6–26.9) (*p* < 0.0003). However the medians of each group and overall median of the two groups were all within the vitamin D insufficient range.

**Fig. 1:**
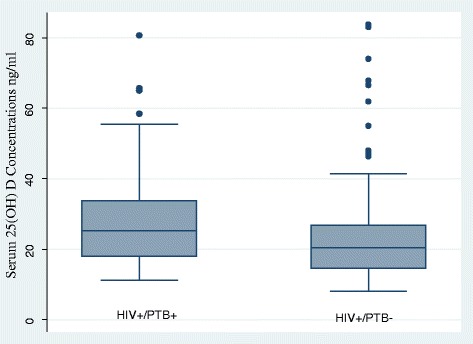
Box and whisker plot showing the distribution of serum vitamin D concentrations by PTB status. Median serum 25(OH) D concentrations were significantly higher in HIV^+^/PTB^+^ group compared to HIV^+^/PTB^−^ group (*p* = 0.0003). Key: HIV+ = Human Immunodeficiency Virus positive, PTB+ = Pulmonary Tuberculosis positive, PTB- = Pulmonary Tuberculosis negative

Distribution of serum 25(OH) D concentrations by PTB status is further illustrated in Fig. 1.

An overall 40.9% (95% CI: 35.1–46.8) were vitamin D deficient, 31% (95% CI: 25.7–36.62) were vitamin D insufficient and 28.2% (95% CI: 23.0–33.8) had sufficient concentrations. Only 1.8% (95% CI: 0.6–4.1) had severe VDD and all were HIV^+^/PTB^−^. Prevalence of VDD at 33.8% (95% CI:26.2–42.1) was significantly lower in the HIV^+^/PTB^+^ compared to HIV^+^/PTB^−^ group (*p* = 0.014). The HIV^+^/PTB^−^ group had a significantly lower proportion of patients with optimal 25(OH) D concentration compared to HIV^+^/PTB^+^ group (*p* = 0.016).

### Correlates of serum 25(OH) D levels

The Wilcoxon rank*-*sum test was used to compare median 25(OH) D concentrations by age, gender, CD4+ T-lymphocyte count, body mass index (BMI), efavirenz (EFV) based cART regimen and cART status. Univariate and multivariate logistic regression analysis were also conducted to ascertain the influence of the same variables on serum vitamin D status (Table 3). For logistic regression analysis, dichotomies of optimal/suboptimal and deficient/not deficient serum vitamin D status were evaluated but only results for suboptimal/optimal category are presented in Table 3.

**Table 3:** Correlates of serum 25(OH) D levels

Variable	Serum 25(OH) D ng/ml Median(IQR)	*p*-value	Univariate Odds ratio (95%CI)	Multivariate Odds ratio (95%CI)
Gender				0.91(0.49–1.62)
Male	20.65 (15.0–30.3)	0.013	Referent	
Female	23.3 (17.9–32.4)		0.84 (0.49–1.42)	
Age				1.32(0.40–2.09)
≤ 50 years	18.91 (15.6–27.5)	0.142	Referent	
> 50 years	22.63 (16.6–32.9)		1.42 (0.7–2.9)	
CD4 Count				0.36 (0.11–1.17)
< 200/μL	22.65 (16.6–32.9)	0.127	0.69 (0.4–1.3)	
≥ 200	19.98 (15.6–28.8)		Referent	
BMI				0.61(0.27–1.38)
≤ 18.5	22.27 (16.6–33.6)	0.643	0.84 (0.5–1.5)	
> 18.5	21.98 (16.4–30.9)		Referent	
EFV-Based Regimen				0.87(0.32–2.34)
Yes	20.50 (15.1–33.1)	0.664	Referent	
No	21.81 (16.6–30.9)		0.97 (0.4–2.3)	
cART status				0.90(0.49–1.62)
Yes	20.84 (15.6–33.0)	0.244	Referent	
No	22.97 (16.9–30.6)		1.04 (0.6–1.8)	

Key: *IQR* interquartile range, *CI* confidence interval, *BMI* Body Mass Index, *EFV* efavirenz, *cART* combination antiretroviral therapy. *p*-values of ≤ 0.05 were considered statistically significant

Female patients had significantly higher median serum 25(OH) D concentration; 23.3 ng/ml (IQR 17.9–32.4) compared to males; 20.65 ng/ml (IQR15.0–30.3) (*p* = 0.013). Age > 50 years, CD4 < 200/μL, BMI ≤ 18.5, being cART-naive and being on non-EFV based- regimen were associated with marginally higher median serum 25(OH) D concentrations though none of these achieved statistical significance. None of these variables was associated with higher odds of vitamin D insufficiency or deficiency in univariate or multivariate logistic regression analysis (Table 3). Further univariate and multivariate logistic regression was also conducted using the same predictor variables but only for the HIV^+^/PTB^+^ patients. Still none of these variables were a significant predictor of vitamin D status.

### Median 25(OH) D concentrations and PTB-related outcomes

Effect of VD status as a dichotomy of optimal or suboptimal on PTB status, duration of hospital stay, cause of death and survival at 8 weeks follow-up post enrolment, was determined using univariate logistic regression. A multivariate logistic regression model incorporating each of PTB status, duration of hospital stay, cause of death and survival at 8 weeks follow-up post enrolment in turn as dependent variables and VD status (≥30 or < 30 ng/ml), gender, age (≥50 or < 50 years), CD4 cell count (≥200 or < 200 cells/μL) and cART status (experienced or naïve) as the explanatory variables was fitted. The OR (95% CI) derived from logistic regression analyses of vitamin D status as an explanatory variable for each of the given PTB-related outcomes adjusted for the other explanatory variables are presented in Table 4.

**Table 4:** Association of serum 25(OH) D concentrations and outcomes in HIV-infected TB suspects

Variable	Serum 25(OH) D ng/ml Median(IQR)	*p*-value	Univariate Odds ratio (95%CI)	Multivariate Odds ratio^b^ (95%CI)
PTB status *n* = 284				1.84 (1.1–3.2)
1. PTB^+^ *n* = 145	25.3 (18.0–33.7)	0.0003	1.91 (1.1–3.2)	
2. PTB^−^ *n* = 139	20.4 (14.6–26.9)		Referent	
8-week outcome *n* = 265^a^				0.90 (0.5–1.7)
1. Alive *n* = 190	21.91 (16.9–31.1)	0.767	Referent	
2. Deceased *n* = 75	21.97 (15.8–31.7)		0.93 (0.5–1.7)	
Cause of Death *n* = 75				4.25 (1.1–16.2)
1. PTB *n* = 49	23.28 (16.6–34.8)	0.059	3.19(0.9–10.7)	
2. Other	18.47 (15.0–29.4)		Referent	
Hospital Stay *n* = 245^a^				0.87 (0.5–1.6)
1. ≤7 days *n* = 179	22.14 (17.0–30.7)	0.740	Referent	
2. > 7 days	22.10 (14.5–35.7)		0.89 (0.5–1.7)	

Key: *IQR* interquartile range, *CI* confidence interval, *PTB* pulmonary tuberculosis, *TB* tuberculosis. *p*-values of ≤ 0.05 were considered statistically significant. ^a^some lost to follow up or incomplete data in records and ^b^OR adjusted for binaries of gender, age (≥50 or < 50 years), CD4 cell count (≥200 or < 200 cells/μL) and cART status (experienced or naïve)

In univariate analysis, patients with optimum serum 25(OH) D concentrations were 1.9 times (OR 1.91; 95% CI 1.1–3.2) more likely to be PTB^+^ compared to those with serum 25(OH) D concentrations < 30 ng/ml. The OR decreased to 1.82 (95% CI 1.1–2.9) when a serum 25(OH) D concentration cut-off of 20 ng/ml was used.

During follow-up, 75 (28.3%) patients died whilst 19 (6.7%) had incomplete data of whom 4(1.4%) were lost to follow up. Those with incomplete mortality data had median serum 25(OH) D of 25.9 ng/ml (IQR18.9–32.4). Of the 75 deceased patients, 49(65.3%) died from a PTB-related cause. Median serum 25(OH) D concentration of patients who died from a PTB-related cause were higher but not significantly so compared to that of those who died from other causes (*p* = 0.059). Similarly, in univariate analysis patients that died from a PTB-related cause were 3.2 times more likely to have serum 25(OH) D levels ≥30 ng/ml compared to those that died from non-PTB related causes, but the difference was not statistically significant (OR3.19; 95% CI 0.9–10.7). None of the explanatory variables (VD status, gender, age, CD4 cell count or cART status) for the cause of death were statistically significant in univariate logistic regression. However in multivariate analysis, patients that died from a PTB-related cause were 4.3 times more likely to have serum 25(OH) D concentration above ≥30 ng/ml; OR 4.25 (95% CI1.1–16.2). Stepwise logistic regression analysis for predictors of cause of PTB related death, fitted a model with gender (OR 0.5; 95%CI 0.2–1.4), cART status (OR 0.39; 95%CI 0.1–1.1) and VD status (OR 3.89; 95%CI 1.1–13.8).

The patients that were recorded as deceased (*n* = 75) at the 8-week follow up time point were re-stratified by initial laboratory based PTB diagnosis. A statistically significantly higher median serum 25(OH) D concentration 24.92 ng/ml (IQR 16.7–34.8) was observed in the PTB^+^ group (*n* = 38) compared to the PTB^−^ group (*n* = 37) with 25(OH) D of 17.58 ng/ml (IQR 14.6–27.1); *p* = 0.026.

No significant differences were observed in median serum 25(OH) D concentrations between patients that died and those that were alive (*p* = 0.77) or those hospitalised for ≥7 days and those hospitalised for shorter durations (*p* = 0.74) after the 8 weeks follow up period.

## Discussion

Our results contribute to a small but growing pool of studies that refute the commonly accepted hypothesis that lower serum 25(OH) D concentrations are associated with PTB [10, 15, 16, 19, 22]. In the present study, median serum 25(OH) D concentrations for both the HIV^+^/PTB^+^ and HIV^+^/PTB^−^ groups were above the threshold for VDD but still below the cut-off for optimum serum 25(OH) D concentration. In addition, we observed significantly higher proportion of patients with optimum 25(OH) D concentrations in the HIV^+^/PTB^+^ group and significantly higher proportion of VDD patients in the HIV^+^/PTB^−^ group.

Furthermore, patients with serum 25(OH) D concentrations above the optimum threshold were twice more likely to be PTB^+^ compared to those with lower concentrations. Surprisingly, we observed no significant association between vitamin D status and all-cause mortality although in multivariate logistic regression analysis patients that died from a PTB-related cause were 4.3 times more likely to have serum 25(OH) D levels ≥30 ng/ml compared to those that died from non-PTB related causes*.*

Our findings though, are in discordance with the generally implied role of vitamin D in mycobacterial immunity that has been suggested by in vitro studies. In vitro studies suggest a role of 25(OH) D in control of MTB infection and by deduction, increased susceptibility in individuals with insufficient 25(OH) D levels. In the present study, HIV^+^/ PTB^+^ patients had insufficient but significantly higher median serum 25(OH) D concentrations compared to HIV^+^/PTB^−^ individuals.

Our observation of higher median serum 25(OH) D levels in HIV^+^/ PTB^+^ participants compared to HIV^+^/ PTB^−^ patients whilst unexpected could be a consequence of active PTB and HIV-infection modulation. MTB and HIV-1gp120 peptides stimulate toll-like receptors 1/2 which in turn have been reported to induce CYP27B1 expression in infected macrophages in vitro [7] We speculate that if the same process occurs in vivo, the resultant accelerated production of 1,25(OH)D might in turn lead to increased mobilisation of 25(OH) D from adipose tissue and consequent elevation in circulation. Alternatively, HIV induced chronic inflammation characterised by tumour necrosis factor-alpha (TNF-α) overproduction, may result in renal 1*α*-hydroxylase impairment [23] leading to reduced parathyroid stimulatory effect on 1,25(OH)2D production thereby leading to accumulation of 25(OH) D in circulation.

We were unable to verify the assertion by Zeng et al.*,* who in a meta-analysis concluded that only severe VDD (< 10 ng/ml) was associated with significant risk of active TB [11]. Five patients were severely vitamin D deficient in our study and interestingly all were PTB^−^ lending support to our observation of lack of association of VDD with PTB albeit the number is small.

The overall 40.9% prevalence of VDD in our study falls within the range of 29–73% reported in literature [24]. Although only an overall 28.2% of the patients had optimal serum 25(OH) D levels, the majority of these were HIV^+^/PTB^+^. In concordance with findings from a study conducted in Uganda [25], 66.2% of HIV^+^/ PTB^+^ patients had serum 25(OH) D levels above 20 ng/ml but this is sharply contrasted by a study from Cape Town South Africa [26] that reported only 14% of HIV^+^/ PTB^+^ patients with concentrations above 20 ng/ml. Such latitudinal-related differences in relative proportions of individuals with insufficient 25(OH) D concentrations among Black Africans from different countries could be attributed to the solar zenith angle that influences the efficiency of ultra violet induced cutaneous synthesis of vitamin D [27].

### Correlates of serum vitamin D concentration

Females had significantly higher median serum 25(OH) D concentrations compared to males contrary to reports of association of male gender with higher serum vitamin D concentrations [28]. Other studies reported no significant differences by gender [29]. We speculate that higher median serum 25(OH) D concentrations in females might have been associated with wasting, the hallmark of both PTB and HIV [30]. Female patients in the present study had marginally higher BMI compared to males. The higher adiposity associated with lower serum 25(OH) D concentrations, may however imply more vitamin D being stored in adipose tissue hence enhanced release during wasting associated with HIV infection and/or active PTB.

Age, CD4+ T-lymphocyte count, BMI, cART status and EFV-based cART regimen were not associated with vitamin D status in univariate or multivariate analysis. Older age has been reported as a risk factor for VDD in an HIV-infected population [31] due to reduced dermal efficiency to synthesize vitamin D [32]. Our findings of no age association may be due to the relatively younger median age of our study patients.

Patients on cART had marginally insignificantly lower median 25(OH) D levels compared to cART-naïve. The association between VDD and cART is controversial. Although VDD has been associated with cART [29] other studies have reported lack of association [8] whilst others reported higher 25(OH) D levels in those on cART [33]. The modulation of vitamin D status by cART has largely been attributed to the effects of protease inhibitors and certain non-nucleoside reverse transcriptase inhibitors (NNRTIs) on vitamin D metabolism.

Similar to findings from another study [34], patients on an EFV-based cART regimen in the present study had marginally insignificantly lower median 25(OH) D levels. NNRTIs notably EFV, have been reported to accelerate 25(OH) D catabolism through the induction of the enzyme CYP24 which converts both 25(OH) D and the active form of vitamin D, 1,25(OH)D, to inactive metabolites [35]. EFV has also been reported to reduce the expression of cytochrome CYP2R1, which hydroxylates vitamin D3 and vitamin D2, an initial step in vitamin D activation [36]. Although the relatively shorter median duration since cART initiation in our study may plausibly contribute to the differences, the possibility of racial differences in the response to EFV cannot be ruled out. Indeed, lack of response to EFV has been reported for non-Caucasian patients [37]. We however, note the possible contribution of diminished power to detect any difference given the limited number of participants on cART and also those on an EFV-based regimen.

### 8-week mortality and 25 (OH) D concentrations

Hypovitaminosis D has been associated with poorer disease outcomes and higher risk of all-cause mortality in the general population as well as in both cART-experienced and naïve HIV-infected individuals [8, 31, 38]. In the present study, median 25(OH) D concentrations were not significantly different between patients that died within the 8-week follow up period and those that were still alive. In multivariate logistic regression analysis, patients that died from a PTB-related cause within 8 weeks had significantly higher odds of having optimal vitamin D status compared to those that were alive. This observation is concordant with the other findings reported in this study and could be explained in a similar manner.

We observed no significant difference in median 25(OH) D concentrations between patients admitted into hospital for at least 7 days and those that were admitted for longer. In concordance with current findings, Sherwood et al. reported no significant associations between 25(OH) D levels with mortality or length of hospitalisation [39]. Similarly, a study conducted in Tanzania observed no relationship between all-cause mortality and dichotomous outcomes of insufficient versus sufficient serum 25(OH) D concentrations although those with sufficient levels had a lower risk of death [11].

### Limitations

The limitations of our study include (i) the cross sectional study design which precludes inference of causal relationships between serum 25(OH) D and PTB, (ii) possibility of reverse causation as 25(OH) D levels were measured when patients were already HIV and/or MTB infected, thus the temporal relationship between infection and vitamin D status could therefore not be established, (iii) stratification of patients into such substrata as cART regimen and cause of death could have diminished statistical power to detect any effect modification and (iv)the lack of consensus on the definitions of vitamin D status in literature also compromised the interpretation of findings across different studies.

Despite these limitations, our study has some strengths. The study sample size was relatively large and although unmatched for gender, age, proportion on cART, BMI and duration on cART, there were no significant differences between the two groups thus giving credence to our findings.

## Conclusions

Our findings though at variance with the generally accepted association between serum vitamin D status and PTB, are in concordance with findings from a few other studies that report an association between high serum 25(OH) D concentrations and risk of PTB. It is possible that the reason for this observed variation could be genetic given the complex and diverse actions of vitamin D. Thus, genetic polymorphisms in the vitamin D receptor, or in the multiple enzymes involved in vitamin D metabolism, remain attractive candidates for further study.

## Abbreviations

25(OH) D: 25 Hydroxyvitamin D
BMI: Body Mass Index
cART: Combination anti-retroviral therapy
CD4: Cluster of differentiation 4
CI: Confidence interval
EFV: Efavirenz
HIV: Human immunodeficiency virus
IQR: Interquartile range
LAM: Lipoarabinomannan
MRCZ: Medical research council of Zimbabwe
MTB: Mycobacterial tuberculosis
NNRTIs: Non-nucleoside reverse transcriptase inhibitors
OR: Odds ratio
PTB: Pulmonary tuberculosis
RCT: Randomised controlled trial
TB: Tuberculosis
VDD: Vitamin D deficiency
Xpert MTB RIF: GeneXpert *Mycobacteria tuberculosis* rifampicin
